# Chronic breast abscess due to *Mycobacterium fortuitum*: a case report

**DOI:** 10.1186/1752-1947-5-188

**Published:** 2011-05-18

**Authors:** Dibendu Betal, Fiona A MacNeill

**Affiliations:** 1 Academic Surgery (Breast Unit), Royal Marsden NHS Foundation Trust, Fulham Road, London, SW3 6JJ, UK

## Abstract

**Introduction:**

*Mycobacterium fortuitum *is a rapidly growing group of nontuberculous mycobacteria more common in patients with genetic or acquired causes of immune deficiency. There have been few published reports of *Mycobacterium fortuitum *associated with breast infections mainly associated with breast implant and reconstructive surgery.

**Case presentation:**

We report a case of a 51-year-old Caucasian woman who presented to our one-stop breast clinic with a two-week history of left breast swelling and tenderness. Following triple assessment and subsequent incision and drainage of a breast abscess, the patient was diagnosed with *Mycobacterium fortuitum *and treated with antibiotic therapy and surgical debridement.

**Conclusion:**

This is a rare case of a spontaneous breast abscess secondary to *Mycobacterium fortuitum *infection. Recommended treatment is long-term antibacterial therapy and surgical debridement for extensive infection or when implants are involved.

## Introduction

*Mycobacterium fortuitum *group is a rapidly growing group of nontuberculous mycobacteria more common in patients with genetic or acquired causes of immune deficiency. There have been few published reports of *Mycobacterium fortuitum *infection in connection with breast infections mainly associated with breast implant and reconstructive surgery. We describe a rare case of a spontaneous breast abscess secondary to *Mycobacterium fortuitum *infection. Recommended treatment is long-term antibacterial therapy and surgical debridement for extensive infection or when implants are involved.

## Case presentation

A 51-year-old Caucasian woman, a professional musician, was referred to our one-stop breast clinic with a two-week history of left breast swelling and tenderness that started prior to her most recent menstrual period. She menstruated regularly and had no previous history of breast disease, and her grandmother had been diagnosed with breast cancer at age 78 years. She had an eight-year-old daughter who was breast-fed for two years and was a nonsmoker with no significant medical history. Preceding presentation there was no history of foreign travel, she was not taking any medications, and she had experienced no trauma to the breast.

The clinical examination revealed redness, induration, and swelling in the upper half of the breast extending to the nipple. There was no nipple discharge, lymphadenopathy, or pyrexia. Her mammogram was normal (M1), and ultrasound revealed nonspecific inflammatory changes with no focal lesions (U3). The clinical impression was of mastitis, but the picture was not typical of periductal mastitis, which is more common in younger women and smokers, nor was it typical of lactational mastitis in breast-feeding women.

A core biopsy demonstrated chronic mastitis (B2) that showed mixed inflammatory lobulitis with fat necrosis and fibrosis, but no vasculitis. No microcalcification, ductal carcinoma *in situ*, or invasive malignancy was identified. Her blood tests (full blood count, renal function, liver function, thyroid function, and blood glucose) were all within normal limits.

She was treated initially with oral flucloxacillin 500 mg four times daily and then switched to co-amoxiclav 625 mg three times daily. After the core biopsy, her breast appearance deteriorated, with worsening swelling, induration, cellulitis, and discharging sinus despite two-week oral antibiotic treatment. Fluid from the sinus was cultured, and there was no bacterial growth or any evidence of methicillin-resistant *Staphylococcus aureus*. The microbiologist at our clinic recommended intravenous antibiotic therapy; however, because of the patient's professional and social commitments, it was not practical for her to be admitted as an inpatient. She was commenced on a higher dose of oral flucloxacillin, 1 g three times daily.

Three weeks after her initial presentation a deep collection became apparent, with pointing on the skin surface. Repeat ultrasound of the left breast confirmed two loculated collections. She was not keen on undergoing any surgical intervention, so outpatient incision and drainage were performed. Specimens were sent for routine bacteriology, acid-fast bacteria stain, Gram stain, and fungal stains and culture. Her pus culture was found to be sterile on routine bacterial culture, so the possibility of an atypical infection such as mycobacterium process was considered and she was started on intravenous flucloxacillin 500 mg four times daily and oral rifampicin 300 mg twice daily.

After another two weeks of treatment, the cellulitis in her left breast had improved, but the breast remained swollen, with multiple sinuses near the nipple. Further ultrasound of the left breast demonstrated multiloculated abscesses. Five weeks after her initial presentation, the patient underwent surgical incision and drainage through the inframammary crease with placement of a small drain to allow gravity-dependent drainage. Repeat histology showed granulomata formation and chronic mastitis with no evidence of malignancy. The initial differential diagnosis included nocardia or *Actinomyces *infection. Subsequent submitted specimens identified *Mycobacterium fortuitum *complex as the causative agent.

After eight weeks of antibiotic therapy with doxycyline 100 mg twice daily and ciprofloxacin 500 mg twice daily, her breast improved clinically. The patient had no further recurrence of the infection and was discharged from our outpatient clinic six months later.

## Discussion

*Mycobacterium fortuitum *is a rapidly growing group of mycobacteria not encompassing *Mycobacterium tuberculosis*, which has been implicated as a cause of pulmonary, soft tissue, and disseminated infections. *Mycobacterium fortuitum *is present in natural and processed water sources as well as in sewage and soil. It may show clinical signs following trauma or surgery after contamination of the wound, medical device implantation, and injection site abscesses. Disseminated disease is usually seen in patients who are immunocompromised, such as individuals who are human immunodeficiency virus-positive or have undergone a course of chemotherapy or corticosteroid treatment [1]. Immune deficiency may be genetic as a result of defects in the interleukin-12/interferon-γ axis. The true incidence of *Mycobacterium fortuitum *infection is unknown, but it has been estimated to be between four to six cases per one million people [2].

There have been few published reports of *Mycobacterium fortuitum *associated with breast infections. There has been only one published report of spontaneous breast abscess due to *Mycobacterium fortuitum *[3] and another report following nipple piercing [4]. The majority of papers have reported an association following breast implant and reconstructive surgery [5-8].

If Mycobacterium complex is suspected as the source of infection, antibacterial therapy should be commenced. Previous publications regarding first-line antimicrobial therapy for *Mycobacterium fortuitum *infection have recommended amikacin, inipenem, fluoroquinolones, cefoxitin, sulfonamides, and linezolid with variable sensitivity to doxycycline and clarithromycin [9-12]. Many reports have suggested success with single-agent treatment, although combination therapy is recommended. However, no standard duration of therapy is reported, and treatment may last up to six months or more [10,11].

Ultimately, the only cure for *Mycobacterium fortuitum *infection may be surgical debridement, especially if the infected area is extensive and the infection involves an implant, which is normally removed [5-8].

## Conclusion

This case highlights an unusual presentation of spontaneous breast infection. If there is no improvement of breast infections despite standard antibiotic therapy, atypical mycobacteria need to be considered as the causative pathogen. Recommended treatment is long-course antibacterial therapy and possible surgical debridement if the area is extensive and implants are involved.

## Consent

Written informed consent was obtained from the patient for publication of this case report and any accompanying images. A copy of the written consent is available for review by the Editor-in-Chief of this journal.

## Competing interests

The authors declare that they have no competing interests.

## Authors' contributions

DB wrote the paper, and FAM performed surgical procedure. Both authors read and approved the final manuscript.
